# Skin-brain dialogue in auto-inflammatory diseases: A new route to biomarkers?

**DOI:** 10.1016/j.bbih.2024.100906

**Published:** 2024-11-09

**Authors:** S. Matar, S. Aractingi, R. Gaillard, A.-C. Petit

**Affiliations:** a Dermatology Department, Hôpital Cochin, AP-HP.Centre-Université Paris Cite, Paris, France; b Université Paris Cité, CNRS, UMR 8104, INSERM, U1016, Institut Cochin, Cutaneous Biology Lab, Paris, France; c Pôle Hospitalo-Universitaire Psychiatrie Paris 15, GHU Paris Psychiatrie et Neurosciences, Hôpital Sainte-Anne, Paris, France; d Université Paris Cité, Paris, France; e Institut Pasteur, Perception and Memory Unit, CNRS UMR, 3571, Paris, France

**Keywords:** Autoinflammatory disease, Skin-brain axis, Psychoneuroimmunology, Neuro-immune-endocrine-cutaneous system, Biomarker

## Abstract

Autoinflammatory diseases (AID) are rare systemic inflammatory disorders due to monogenic or polygenic dysfunction of innate immunity. They affect many organs including the brain and the skin. The spectrum of these diseases has been rapidly expanding recently due to newly developed diagnostic tools. The neuro-immuno-endocrine-cutaneous interactions play an important role in the pathophysiology of these diseases. The skin-brain interplay is not fully investigated in AID and evidence supporting bidirectional communication is examined. This article provides an overview of the current state of the art in the pathophysiology of AID with cutaneous and psychiatric manifestations. Elucidating the neuro-immuno-endocrine-cutaneous dysregulation underlying pathophysiology of AID is promising for determining future biomarkers and therapeutic options.

## 1 Introduction

Since autoinflammation was first defined by McDermott et al., in 1999 (McDermott et al., 1999), numerous definitions and classifications of autoinflammatory diseases (AID) have been presented. Due to innate immune system defects, AID are characterized by spontaneous inflammation with elevated inflammatory blood biomarkers but without autoantibodies or antigen-specific T cells (Georgin-Lavialle et al., 2019). The spectrum of monogenic AID includes now inflammasomopathies such as pyrinopathies, cryopyrinopathies, PSTPIP1-associated autoinflammatory diseases (PAID), and monogenic diseases due to deficiency of cytokines receptors antagonists or macrophage activation (see Table 1) (Georgin-Lavialle et al., 2019). Due to advance in genetic and immunology fields, new AID are often added such as Vacuoles, E1 enzyme, X-linked autoinflammatory somatic syndrome (VEXAS) which was recently described and belongs to ubiquitinopathies and NFκB disorders (see Table 1) (Ashari et al., 2023). Inflammasomopathies involve abnormal activation of inflammasomes leading to excessive production of pro-inflammatory cytokines like interleukin-1β (IL-1β), IL-18, IL-6, and Tumor Necrosis Factor-α (TNFα) (Georgin-Lavialle et al., 2019; Moltrasio et al., 2022). NOD-like receptor family, pyrin domain containing 3 (NLRP3) mutations are specifically associated with cryopyrinopathies, causing high IL-1β levels that trigger immune activation (Georgin-Lavialle et al., 2019). Elevated IL-6 and TNFα contribute to a persistent inflammatory state, and TNF pathway anomalies are common in certain AID cases. IL-18 also plays a role, particularly in macrophage activation syndrome (MAS) (Musters et al., 2015b; Blackstone et al., 2023).

**Table 1:** Clinical and pathophysiological characteristics of main monogenic AID with dermatological and neuropsychiatric manifestations. Nearly all AID have cutaneous manifestations but for the sake of simplicity our table is not exhaustive and focus on AID with frequent skin involvement and associated neurological or psychiatric symptoms. Commonly used biological biomarkers to monitor disease activity and treatment response are CBC (Complete Blood Count) and CRP (C-reactive protein), that are non-specific. Recently authors suggest serum cytokines level assessment but they also lack specificity. AR: Autosomal recessive, AD: Autosomal dominant, F: Female, M: Male, WBC: White Blood Cells, Neutro: neutrophils, CSF: Cerebrospinal Fluid, MAS: Macrophage Activation Syndrome, NSAIDs: Non-steroidal anti-inflammatory drugs, SAA: serum amyloid A protein, HSM: Hepatosplenomegaly, LAD: Lymphadenopathy, i.e.: id est, NET: Neutrophil Extracellular Traps, Tie: Tyrosine-protein kinase receptor, sFlt-1: soluble Vascular Endothelial Growth Factor receptor-1, sRAGE: soluble isoform of a Receptor for Advanced Glycation End products, sICAM: Circulating intercellular adhesion molecule 1, TNFr: TNF receptor, JAKI: JAK inhibitors, TKI: Tyrosine Kinase inhibitors.

Disease	Genetic and Epidemiological Characteristics	Pathophysiologic mechanism	Skin manifestations	Neuro/psychiatric manifestations	Suggested biomarkers of AID	Systemic treatment
IL-1 related AIDs						
Inflammasomopathies						
**Pyrinopathies**						
Familial Mediterranean Fever (FMF)	MEFV [AR]Pyrin (marenostrin) M/F: 1.5–2:1 Most common AID 1:200–1000 people in populations originating from the mediterranean region	Dyregulation of inflammasomes leading to high levels of IL1 and IL18	Erysipelas-like skin lesions on distal extremities ± Henoch- Schonlein purpura Cutaneous periarteritis nodosa	Headache (47.26%) Migraine (6.1%) Vertigo (26.7%) Paresthesia (23.2%) Tremor (17%) Disorientation (12.9%) Breath-holding (27.4%) Migraine (6.1%) Syncope (2.6%) Epilepsy (2.3%) Febrile seizure (1%) Ataxia (1.6%) Intracranial hypertension Chronic aseptic meningitis. Depression, anxiety, sleep disturbances, suicidal ideation	CRP CBC (↑WBC with ↑neutrophils) Inflammatory cytokines S100A12, S100A8/A9 Ferritin Serum asprosin level	ColchicineNSAIDsAnalgesicsAnti-TNF-α that can have significant antidepressive effectsAnakinra Positive reported results with antidepressant SSRIs
Hyper IgD Syndrome (HIDS)	MVK [AR]Mevalonate kinase M/F: 3:2 1:50,000 to 1:5000 prevalent mainly in northern Europe	Mevalonate kinase is involvedin cholesterol biosynthesis	Erythematous macules Papules and nodules Urticaria Aphthous oral/genitalulcers	Headaches (41%) Ataxia and developmental delay in less than 1%. Chronic aseptic meningitis Stroke Attention deficit and low intellectual performance Eating disorder	CRP. CBC (↑WBC with ↑neutrophils) Inflammatory cytokines. Serum IgD level	PrednisoneIVIGColchicineCyclosporine AStatinsAnti-TNF-αAnakinra
**PAID for PSTPIP1-associated autoinflammatory diseases**						
PAPA syndrome	PSTPIP1/CD2BP1[AD]M/F:2.2 Rare: around 200 patients worldwide	CD2BP1 can bind to inflammasome pyrin	Pyoderma gangrenosumAcne	Cerebral arterial vasculopathy Cerebral artery aneurysm	CRP CBC (↑WBC with ↑Neutro) Inflammatory cytokines	Tetracycline/Isotretinoin foracnePrednisoneAnti-TNF-αAnakinra Reports on efficacy of IL17 inhibitors
PAMI syndrome (PSTPiP1-Associated Myeloid-related-protein I		Increase in the synthesis of a pro-inflammatory alarmin, MRP8/14 (myeloid-related-protein) protein, also known as calprotectin or S100A8/A9	Pyoderma gangrenosum (50%) Ulcerated and/or necrotic lesions (30%) Acne and/or pustular rash (20–25%)	Mental retardation Rarely cerebral vasculitis Axial hypotonia Irritability		
PASH syndrome		Dysregulation of proteins of the inflammasome complex or proteins that regulate its function	Pyoderma gangrenosumAcne HS	Depression Anxiety		
PAPASH syndrome			Pyoderma gangrenosum Acne HS			
PAC syndrome			Recurrent skin ulcerations ± Pyoderma gangrenosum. Severe pustular rash Acne			
PAPA-like syndrome			Pyoderma gangrenosum and acne-like syndrome Mouth ulcers			
**Cryopyrinopathies (Cryopyrin Associated Periodic Syndrome-CAPS) or NLRP3associated Auto-Inflammatory Disease (NRLP3-AID)**						
Familial ColdAutoinflammatorySyndrome (FCAS)	CIAS1/NLRP3 [AD]Cryopyrin, FCAS M/F: 2: 1 MWS M/F:1:1.5 CINCA: M/F: 1:3.5 1–2 : 1 000 000 people	Dyregulation of inflammasomes, leading to high levels of IL1 and IL18	Pink figurate patches orred macules/papules Not true urticaria	Subdural hemorrage Cerebral infarction/hemorrage Intracranial hypertension Chronic aseptic meningitis Sensorineural hearing loss. Optic neuritis Brain atrophy Seizure Reported risk of multiple sclerosis Mental retardation Depression Anxiety Low quality of life: risk of social isolation, low productivity and absenteism from school or work	CRP CBC (↑WBC with ↑neutrophils) Inflammatory cytokines S100A12, S100A8/A9 CSF cytokines level in aseptic meningitis	AnakinraRilonaceptCanakinumabThalidomide in CINCA/NOMID
Muckle-WellsSyndrome (MWS) or urticaria-deafnessamyloidosissyndrome			Urticaria			
Chronic InfantileNeurologicCutaneousArticularSyndrome –Neonatal OnsetMultisystemInflammatorySyndrome(CINCA/NOMID)			Urticaria			
**Other inflammasomopathies (newly established)**						
Diseases related to NLRC4 mutations	NLCR4 [AD]	Dyregulation of inflammasomes, leading to high levels of IL1 and IL18	Unspecific cutaneous rash (70%)	Discussed multiple sclerosis risk Chronic aseptic meningitis Depression	CRP CBC (↑WBC with ↑neutrophils) Inflammatory cytokines mainly high IL-18	CorticosteroidsAnakinra Ciclosporin Positive reports on efficacy of anti-IL18 and anti-IFN gamma agents in life-threatening cases
			Cold urticaria Erythematous nodes Painful lymphohistiocytic panniculitis			Abstention or symptomatic treatment with NSAIDs
Periodic fever associated with NLRP 12 mutations	NLRP12 <1:1 000 000 people	Dyregulation of inflammasomes, leading to high levels of IL1 and IL18 Suggested modulation of IL4 producing T cells	Cold-induced urticaria	Headache Sensorineural hearing loss Optic neuritis Depression	CRP CBC (↑WBC with ↑neutrophils) Inflammatory cytokines	anti-IL1 anti-IL18 under trial
Auto inflammatory syndrome with arthritis and dyskeratosis associated with NLRP1 mutations NAIAD	NLRP1 [AR] or [AD]	Dyregulation of inflammasomes, leading to high levels of IL1 and IL18 Decrease in CD27^+^ memory B cells	Papillary or filiform hyperkeratosis Pseudo-phrynoderma HPV negative condylomas Extended candidiasis	Anxiety Depression	CRP CBC (↑WBC with ↑neutrophils) Inflammatory cytokines mainly high IL-1	anti-TNF anti-IL-1 therapy
**Deficiency of receptors antagonists**						
DIRA (deficiency of the IL1 receptor antagonist)	IL ra [AR]. M/F: 2:1 in some series 1:6300 people	Non counterbalaced action of IL-1	Pustulear dermatitis resembling pustular psoriasis	Possible neurological deficits due to vertebral lesions/instability	CRP CBC (↑WBC with ↑neutrophils) Inflammatory cytokines mainly high IL1	Anakinra
DITRA (deficiency of the IL36 receptor antagonist)	IL 36 ra [AR] M/F: 10:3 <1:1 000 000 people	Non counterbalaced action of IL-36	Early onset of generalized pustular psoriasis	Depression	CRP CBC (↑WBC with ↑neutrophils) Inflammatory cytokines mainly high IL-36	Anakinra partially effective Promising results with ustekinumab (humanized antibody selectively blocking IL-12 and IL-23)
**M1 Macrophage activation**						
ADA2 deficiency (DADA2)	DADA2 [AR] adenosine deaminase 2 M/F:0,91 1:222 000 people	Insufficient or absent ADA2 enzymatic activity leading to a reduction of adenosine conversion into inosine and to an accumulation of adenosine in the extracellular space Chronic neutrophils activation and NETosis dysregulation with spontaneous NETs formation	Non-specific skin rashes and mouth ulcers Cutaneous periarteritis nodosa Reported sweet syndrome (neutrophilic dermatosis/histiocytoid variant) Reported panniculitis	Acute or chronic small-vessels ischemia (deep brain nuclei and/or the brain stem) Transient ischemic attacks Hemorrhagic strokes Anxiety	CRP CBC (↑WBC with ↑neutrophils) Inflammatory cytokines Tie-1, Tie-2 sFlt-1 sRAGE and TNF-α	Anti-TNF therapies recommended for vascular phenotype of DADA2 IV immunoglobulin supplementation if symptomatic immunodeficiency
**Other monogenics AIDs**						
PFAPA syndrome or Marshall’s syndrome	Variants in inflammasome related genes (NLRP3 and MEFV) Possible role of these genes in PFAPA pathogenesis M/F:1.3:1 0.86:10,000 people	Probable inflammasomopathy	Rare truncal erythemaAphthous stomatitis(labial gingiva)	Febrile seizure Separation anxiety and restlessness in children Depression Anxiety	CRP CBC (↑WBC with ↑neutrophils) Inflammatory cytokines Low vitamine D levels Elevated CD64 expression on neutrophils and monocytes Low CD8^+^ T cells in tonsillar germinal centers	PrednisoneCimetidineAnakinraTonsillectomy
CANDLEsyndrome (ChronicAtypicalNeutrophilicDermatosis withLipodystrophy)	PSMB8 [AR]PSMB8 M/F: 60%–500 reported cases	Abnormal functioning of the multicatalytic system proteasome –immunoproteasome	Chronic Atypical Neutrophilic Dermatosis Annular erythematous/violaceousplaqueViolaceous edema around eyelids/lipsPartial lipodystrophy	Seizure Irritability	CRP CBC (↑WBC with ↑neutrophils) Inflammatory cytokines	PrednisoneMethotrexateTacrolimusInfliximabAdalimumab (anti-TNF)AnakinraTocilizumab
Chronic RecurrentMultifocalOsteomyelitis(CRMO)	LPIN2 [AR]Lipin-2 M/F: 1:2 1:160,000 to 1: 2,000,000 people	Variable defects in the TLR4/MAPK/inflammasome signaling cascade Imbalance between pro- and anti-inflammatory cytokine expressions in monocytes	Psoriasis Palmoplantar pustulosis AcneNeutrophilic dermatitis(i.e. Sweet’s syndrome)	Headache Aseptic meningitis Hypertrophic pachymeningitis Mood disorder/Depression Suicidal ideation Eating disorder Anxiety	CRP CBC (↑WBC with ↑neutrophils) Inflammatory cytokines	PrednisoneNSAIDsAnemia:splenectomy + bloodtransfusions
Recurrent fever and polyarthritis with LACC1/FAMIN mutations	LACC1/FAMIN [AR] encodes the Laccase enzyme 16–150: 100,000 people in European countries	Reduced or complete loss of Laccase function, a controller of energy homeostasis in macrophages May promote sterile inflammation	Maculopapular rash	Anxiety Depression	CRP CBC (↑WBC with ↑neutrophils) Inflammatory cytokines	Corticosteroids Adalimumab
Hidradenitis suppurativa associated with nicastrin mutations (NCSTN)	Nicastrin [AD] M/F: 1:2	Nicastrin is a transmembrane protease type glycoprotein acting as a gamma-secretase	Hidradenitis suppurativa: recurrent aseptic abscesses of the large folds (axillar, inguinal, perineal, and anorectal) Pyoderma gangrenosum Acne	Unipolar or bioplar depression Anxiety Schizophrenia Suicide	CRP CBC (↑WBC with ↑neutrophils) Inflammatory cytokines	anti-TNF adalimumab reduces pain and improves depressive symptoms Trial of: anti-IL-1 anti-IL17 anti-IL23
**Ubiquitinopathies/NFκB disorders**						
**NFκB disorder with TNF receptor activation**						
TNF Receptor Associated Periodic Syndrome (TRAPS)	TNFRSF1A [AD]TNF Receptor 1 Most common mutations areR92Q and P46L M/F: 3:1 1:1,000,000 people	Non counterbalaced action of TNF	Erythematous patches Edematous plaques often annular/serpiginous Ecchymotic evolution	Headache Diziness Cerebral infarction/hemorrage Irritability Depression Anxiety	CRP CBC (↑WBC with ↑neutrophils) Inflammatory cytokines Level of circulating TNFr sICAM-1 in TNFreceptor	PrednisoneAnti-TNF-α can improve depressive symptoms IL-1 inhibitors:Anakinra Canakinumab (continuous treatment regimen)
Recurrent fever associated with TNFRSF11A mutations	TNFRSF11A	Increased secretion of cytokines and altered NF-κB signalling	Erythematous patches Edematous plaques often annular/serpiginous Ecchymotic evolution	Discussed Multiple Sclerosis risk	CRP CBC (↑WBC with ↑neutrophils) Inflammatory cytokines	PrednisoneAnti-TNF-α IL-1 inhibitors
**Ubiquinopathies**						
HA20 haploinsufficiency	A20 protein regulates ubiquitination [AD] F/M: 68.6%	Dysregulation of ubiquitination or post-translational recognition and destruction of proteins labeled by the proteolytic complex of the proteasome Negative regulation defects of the NFκB pathway with excessive production of IL1, IL6, TNF (abnormal TNF regulating pathway)	Bipolar aphthae ulcers: Oral aphtae ulcers (88%) Genital aphtae ulcers (68%) Various cutaneous lesions (45%)	Non specified reported neurological symptoms	CRP CBC (↑WBC with ↑neutrophils) Inflammatory cytokines	Colchicine Corticosteroids Immunosuppressants (methotrexate, azathioprine, thalidomide, cyclophosphamide, tofacitinib) Anakinra Tocilizumab Anti-TNF
Vacuoles, E1 enzyme, X-linked autoinflammatory somatic syndrome VEXAS (discovered in 2020)	UBA1 gene on X chromosome [Acquired] (clonal expansion of hematopoietic stem or progenitor cells with acquired mutations in UBA1 gene) > 80% of reported patients are males	Newly established AID UBA1 gene encodes for a key enzyme of the ubiquitylation proteasome system	Skin lesions (83.6%) Non vasculitic: Neutrophilic dermatoses(erythema nodosum, Sweet’s syndrome) Pressure plaques Urticaria Injection site reactions Vasculitic: leukocytoclastic vasculitis	Hearing loss Sensory neuropathy Acute attacks of chronic inflammatory demyelinating polyneuropathy		High-dose glucocorticoids Hypomethylating agents (Azacitidine) are the standard treatment for hig-risk myelodysplastic syndrome Hemopoetic stem cell transplantation JAKI (Ruxolitinib or Baricitinib) can significantly improve mood and quality of life
**NFκB disorder**						
Pytiriasis rubra pilaris syndrome associated with CARD14 mutations CAMPS	CARD14 [AD]	CARD14 gene encode a protein expressed on keratinocytes and capable of activating the NF-KB pathway	Early onset of pytiriasis rubra pilaris rash Extensive erythematous scaly coalescent plaques with islets of healthy skin + follicular papules + palmoplantar keratoderma	Anxiety Depression	CRP CBC (↑WBC with ↑neutrophils) Inflammatory cytokines	Retinoids Cyclosporine Anti-TNF
Blau syndrome or familial juvenile systemicgranulomatosis	NOD2/CARD15[AD]NOD2/CARD15 M/F: 1 < 1:1,000,000 people	NFκB pathway dysregulation	Tapioca grain-like’yellowish to brown-redpinhead-sized papules	Encephalitis Hydrocephalus(reported case) Anxiety Depression	CRP CBC (↑WBC with ↑neutrophils) Inflammatory cytokines S100A12, S100A8/A9	PrednisoneMethotrexateCyclosporine AAnti-TNF-αAnakinra Anti-JAK Tofacitinib
NEMO deleted exon 5 autoinflammatory syndrome NDAS	IKBKG 1:100,000 people	Loss-of-function mutations in IKBKG encoding NF-κB essential modulator (NEMO)	Nodular skin rash Sterile lymphohistiocytic panniculitis	Optic neuritis Chorioretinitis	CRP CBC (↑WBC with ↑neutrophils) Inflammatory cytokines	Corticosteroids JAKI: Baricitinib can significantly improve mood and quality of life Adalimumab
**Other**						
Disabling pansclerotic morphea	STAT4 mutations suggested	Newly established AID Mechanism still unknown	Morphea plaque	Seizure Embarrassment (53%) Anxiety Depression		Glucocorticoids Mycofenolate Mofetil Trials not conclusive on JAKI and TKI Hemopoetic stem cell transplantation

These overproduced cytokines have been widely associated with neuroinflammation (Miller et al., 2017). Recent data shows that blood cytokine network is altered in psychiatric disorders (Goldsmith et al., 2016) and that blood levels of IL-3, IL-6, IL-12, IL-18, soluble IL-2 receptor and TNF-α are significantly altered in depressive patients and could be investigated as biomarkers of depression (Osimo et al., 2020)Zhang et al., 2024. TNFα, IFN-γ, and IL-1β are cytokines that have been shown to activate monoamine reuptake, stimulate the HPA axis, and decrease serotonin synthesis by increasing the activity of indolamine-2,3-dioxygenase (IDO) (Lichtblau et al., 2013). Central TNFα and IL-1β also modulate synaptic plasticity which is a crucial process involved in mood disorders (Khairova et al., 2009).

The same overproduced cytokines have been implicated in skin inflammatory diseases by direct effect on immune cells and keratinocytes or indirect effect on sensory nerves leading to aberrant local neuropeptide production and maintained neurogenic inflammation (Pondeljak and Lugović-Mihić, 2020). Notably, patients with monogenic AID often present with challenging dermatological and psychiatric manifestations concomitant to systemic inflammatory state (see Table 1 and (Shwin et al., 2017; Diprose et al., 2022)).

Recent studies reveal a two-way interaction between the Central Nervous System (CNS) and the immune system, showing highly complex communication, particularly at the level of cerebral barriers (Castellani et al., 2023). Animal data suggest relevant mechanisms for AID: peripheral IL1β can directly activate brain circuitry leading to hypothalamic nuclei activation and corticosterone secretion (Jagot et al., 2023)with immunomodulatory effect; interestingly, IL1β also activates mood-related areas like the locus coeruleus. Key regulatory pathways of neuro-immune communication include the hypothalamus–pituitary–adrenal (HPA) axis (with secretion of glucocorticoids by CNS, such as corticosterone in animals and cortisol in humans) and the autonomic nervous system (ANS), notably the vagus nerve in parasympathetic system, responsible for the inflammatory reflex (Pavlov and Tracey, 2012), and the sympathetic nervous system (SNS), which releases immunosuppressive noradrenaline (NA) and neuropeptides (Haykin & Rolls, 2021). As for the skin, HPA axis hormones trigger cutaneous immunological responses, with skin cells expressing receptors for these peptides and releasing themselves neuropeptides; neuro-immune mechanisms of systemic inflammation can then be locally modulated and/or amplified (Slominski et al., 2015). For example, neuropeptides like Corticotropin-releasing-hormone (CRH) and neurotensin increase skin vascular permeability, worsening stress-related conditions. (Pondeljak and Lugović-Mihić, 2020; Donelan et al., 2006).

The objective of this comprehensive review is to summarize the state of the science on the skin-brain interplay in AID with skin and psychiatric manifestations from a psychoneuroimmunology (PNI) perspective. Specifically, we aim to elucidate the suggested pathophysiological pathways implicating aberrant cytokines and neuropeptides production as well as aberrant skin-brain communication in AID pathogenesis. Furthermore, we discuss emerging and possible future therapeutic approaches targeting underlying inflammatory hyperactivation cascades. We also explore the potential of this neuroimmunological dialogue as a route to biomarker discovery. Our goal is to shed light on the importance of integrating PNI perspectives in the research and treatment of AID with both skin and psychiatric manifestations.

### 1.1 Current knowledge about the psychiatric facet of AID

The effect of stress in AID pathophysiology has rarely been studied. Numerous research has been conducted on the risk variables that lead to Familial Mediterranean Fever (FMF) attacks. Physical and psychological stresses are known to trigger FMF episodes requiring treatment adjustment, while patients improve during stress-free holidays(Korkmaz et al., 2020). The most frequent triggers for attacks were discovered to be social interactions, job interviews, out-of-town trips, and school examinations; moreover, attacks were strongly correlated with family dysfunction and hostility in pediatric FMF patients (Korkmaz et al., 2020). A conditional logistic regression method estimated 70% higher chance of experiencing an FMF episode on the second day after an extra stressful occurrence (Yenokyan and Armenian, 2012). In one trial, individuals with FMF and depression symptoms, resistant to colchicine treatment, had fewer episodes of attack when they took selective serotonin reuptake inhibitor (SSRI) antidepressant drug (Onat et al., 2007). Remarkably, an RNA sequencing analysis conducted on peripheral neutrophils from three FMF patients with psychosocial stress during attacks revealed a considerable overexpression of the stress-related protein regulated in development and DNA damage responses 1 (REDD1) during FMF episodes (Skendros et al., 2017). Psychological stress-related mediators like adrenaline cause REDD1 upregulation, intracellular IL-1β synthesis, and neutrophil extracellular traps release, then increasing inflammation (Skendros et al., 2017). This highlights the role of psychological stress in enhancing the inflammatory loop in AID and further future investigations should better elucidate this psycho-neuro-immunological communication in AID.

On the other hand, there is increasing literature assessing the psychiatric impact of AID in patients. Few small cohorts indicate that patients with AID suffered from significantly altered quality of life, reduced physical activity, and more pain and fatigue compared to matched healthy controls (Rech et al., 2024). A cross-sectional study of patients suffering from FMF showed a higher prevalence of anxiety and depression in patients (4,93%) compared to controls (3,14%) (Lidor et al., 2021). Patients with FMF in a more recent cohort scored anxious and mildly depressed (Kilinc et al., 2024). Compared to healthy children, children and adolescents with FMF have greater sleep issues, and psychiatric symptoms are more common when sleeping disturbances are present (Durcan et al., 2020). It is also well known that patients with autoimmune disorders have a 50% higher risk of anxiety and depression as compared to controls (Sloan et al., 2024). In both disorders, psychiatric symptoms are linked to chronic inflammation and aberrant cytokines production. Recently, a study assessing thirty patients with autoimmune and autoinflammatory diseases showed that overall AID were associated with a positive psychiatric diagnosis (Nudel et al., 2023). Machado et al. have estimated that the prevalence of depression in 40,307 patients with Hidradenitis Suppurativa (HS) was 16.9% (Machado et al., 2019). The prevalence of anxiety in patients with HS can be up to 18% (Caccavale et al., 2023). A higher risk of bipolar disorder (OR 4.7), schizophrenia (3.8) as well as suicide (OR 2) were also reported in patients with HS (Caccavale et al., 2023). A study assessing 34 patients with Chronic Recurrent Multifocal Osteomyelitis (CROM) reported mood disorder, suicidal ideation, eating disorder and anxiety disorder as possible comorbidities (Walsh et al., 2015). Patients with CAPS syndrome can suffer from depression, lower quality of life, anxiety, and risk of social isolation, lower productivity and absenteeism from school or work (Mulders-Manders et al., 2018; Welzel and Kuemmerle-Deschner, 2021). Lower levels of anxiety in patients with AID were linked to better levels of education and medication adherence (Kilinc et al., 2024). Similarly, there was less anxiety in the individuals whose genes were linked to severe illness course, possibly due to better follow-up, compared to those with mild illness that should require specific attention and better management (Kilinc et al., 2024). Future studies should better assess the psychiatric comorbidities in patients with AID, in particular the specifically altered psychiatric dimensions and their response to standard treatments, to improve their quality of life.

### 1.2 Exploring the pathophysiology of skin-brain interaction in AID

**Fig. 1:**
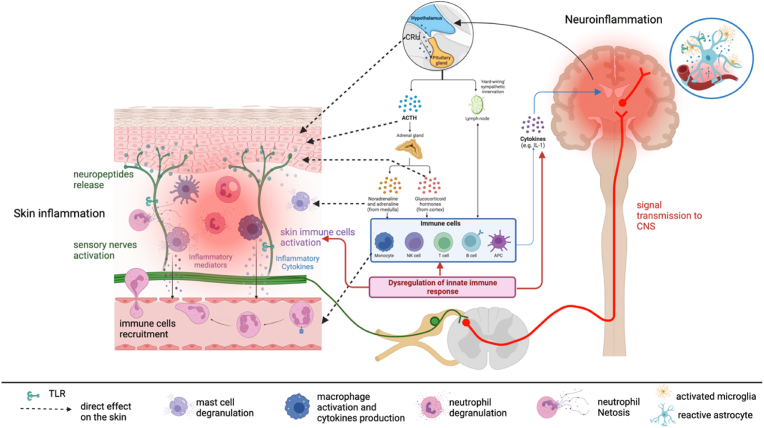
Brain-skin dialogue in AID. In AID, there is a dysregulation of the innate immune system: immune cells (blue frame) produce and secrete cytokines and inflammatory mediators (blue arrows). These mediators additionally stimulate cutaneous immune cells and sensory neurons in the skin. The stimulation of sensory neurons triggers the secretion of neuropeptides, which in turn activate both immune and non-immune cells in the skin. Additionally, these neuropeptides transmit signals to the brain, which receives proinflammatory signals from this nervous route but also through blood route, possibly leading to neuroinflammation in the brain. TLRs found in the central nervous system (CNS), particularly in microglia, play a crucial role in controlling the production of proinflammatory cytokines. In turn, the brain transmits messages to the peripheral nervous system through neuronal or endocrine messengers, specifically by activating the HPA axis and the autonomic nervous system (ANS), particularly the sympathetic nervous system (SNS) which produces noradrenaline (NA) and neuropeptides. The activation of these descent pathways can directly affect the skin inflammation by activating CRH, ACTH and glucocorticoids receptors in skin cells, by stimulating mast cell degranulation through NA signaling or by activating neuropeptides receptors on skin cells. Additionally, it can indirectly contribute to skin inflammation by activating immune cells in peripheral areas and lymph nodes, which are then recruited to the skin and other affected organs. The disruption of the bidirectional interaction between the brain and skin perpetuates the autoinflammatory loop in AID. TLR: Toll-like receptors; HPA: Hypothalamic-Pituitary-Adrenal; CRH: Corticotropin-releasing hormon; ACTH: Adrenocorticotropic hormone.

The pathophysiology of the skin-brain interaction in AID is not yet fully elucidated. It involves a complex interplay between immune dysregulation, sensory neuron activation and neuroinflammation (see Fig. 1). In AID, dysregulation of the innate immune system leads to inflammation and skin damage responsible for cutaneous manifestations of AID (Table 1).

The skin is not only the target of auto-inflammation but it could play an important role in its generation and maintenance. The skin houses various immune cells and is involved in the production and release of inflammatory mediators (Feng et al., 2024). This inflammation triggers the activation of immune cells in the skin, such as mast cells and dendritic cells. These immune cells release pro-inflammatory cytokines and chemokines, which can activate keratinocytes. In turn, keratinocytes cause the epidermis to generate proinflammatory cytokines such IL-1α, IL-6, and IL-8 (Marek-Jozefowicz et al., 2023). The activation of skin immune cells also affects sensory nerves in the skin (Fig. 1). The skin contains myelin-type Aδ fibers, autonomic nerve fibers, unmyelinated C-fibers, and afferent fibers, which are densely distributed across all of the skin's layers. Neuropeptides emitted by nerve fibers can activate cutaneous immune cells and maintain the auto-inflammatory loop. For example, neuropeptides, such as substance P (SP) and calcitonin gene-related peptide (CGRP), are secreted from the epidermal nerve endings, and trigger neurogenic inflammation by activating mast cells, causing degranulation and release of several pro-inflammatory cytokines and vasoactive amines exacerbating inflammation (Feng et al., 2024; Marek-Jozefowicz et al., 2023). In AID, we might assume that patients could have elevated neurotrophins and neuropeptides expression in their skin as already proven in patients with inflammatory skin diseases (Marek-Jozefowicz et al., 2023), but this needs to be properly proven.

The transmission of these various inflammatory signals from the skin to the CNS involves parallel routes (Fig. 1):1) the skin sensory fibers constitute the cutaneous neuroendocrine system and release many neuropeptides that act as blood messengers to the CNS (Marek-Jozefowicz et al., 2023). This can result in pain and neuroinflammation (Feng et al., 2024); 2) the aberrant cytokines produced in the skin can circulate in the blood and enhance neuroinflammation that could enhance psychiatric manifestations; 3) the skin contains a wide variety of cell types that express Toll-like receptors (TLRs) (Feng et al., 2024). Recent studies have suggested that TLRs expressed on cutaneous sensory nerves may play a role in the communication between the skin and the brain in AID (McColl et al., 2016). We can expect that hyperactivation of TLRs on skin sensory nerves in AID facilitates communication between the brain and inflamed skin (Lacagnina et al., 2018). Lastly, it has been proven that cutaneous immune activation is associated with significant immune cells infiltration into the brain that could explain the psychiatric manifestations in AID (McColl et al., 2016).

This cutaneous neurogenic inflammation shows the intricate and dynamic communication network that underpins the neuro-immuno-endocrine-cutaneous axis and can explain the pathophysiologic mechanisms of skin and psychiatric manifestations in AID. Thus, the persistent inflammation, pain perception, and neuroimmune dysfunction seen in AID are caused in part by this dysregulation of the skin-brain communication.

### 1.3 The need of novel biomarkers

Measurements of CRP, erythrocyte sedimentation rate (ESR), serum amyloid A protein (SAA), complete blood count, and type I interferon signatures may be used in systemic monitoring of inflammation, depending on the specific condition (Table 1).

CRP and many serum cytokines levels have been recently suggested as biological biomarkers of psychiatric diseases and skin diseases such as TNF-α, IL-1β, IL-6, and IL-18 (Tan et al., 2024; Eggertsen et al., 2024; Osimo et al., 2020). Serum levels of NLRP3 have also been suggested as biomarkers of major depressive disorder (MDD) (Guo et al., 2024). However, these blood markers are not specific to MDD. For example, significantly elevated levels of CRP, IFN-γ and TNF-α were found in patients with Generalized Anxiety Disorder (GAD) compared with healthy controls (Costello et al., 2019). A combined panel of serum S100 calcium-binding protein B (S100B) neurotrophic factor and cytokines (including IL-1β, IL-2 levels and IL-4) was suggested as diagnostic biomarker for GAD (Shen et al., 2022).

Because of the heterogeneity of patients suffering from mood and anxiety disorders and the lack of identified pathophysiological mechanisms, none of these suggested biomarkers is currently validated in a specific disease.

In the specific context of patients suffering from AID associated with cutaneous and psychiatric manifestations, this search for biomarkers could be particularly informative as it offers a specific pathophysiological framework.

A recent study revealed that patients with FMF have impaired tryptophan metabolism, the serotonin precursor, with a shift towards the kynurenine pathway: they present decreased serum levels of tryptophan and kynurenic acid and increased levels of kynurenine, 3-hydroxykynurenine, quinolinic acid, and kynurenine/tryptophan ratio as compared to controls (Tezcan et al., 2023). It suggests that tryptophan degradation through the kynurenine pathway is increased in patients with FMF. A number of studies have suggested the assessment of serum tryptophan and serotonin metabolism in patients with MDD due to their implication in the pathophysiology of affective disorders (Li et al., 2024). Recently, a potential role of kynurenine activation has been suggested in inflammatory dermatosis such as psoriasis suggesting that this pathway could be the missing link between skin manifestations and depressive symptoms in patients (Stepaniuk et al., 2023). Further investigation of the kynurenine pathway's role in the skin-brain dialogue is needed and serum levels of tryptophan metabolites can be suggested as biomarkers of subclinical inflammation in AID (Tezcan et al., 2023).

The skin-brain dialogue appears early in life. Epidermal integrity assessment by transepidermal water loss or electrical impedance spectroscopy has been assessed in mouse models and humans with autism spectrum disorder indicating skin barrier alterations (Jameson et al., 2023). The skin-brain connection is also gaining interest in neurodegenerative Alzheimer's disease, where patients have less acidic skin pH, reduced skin elasticity (not solely attributable to age) as well as α-synuclein deposition in normal-appearing skin (Klostermeier et al., 2023; Wu et al., 2023). The above findings in neuropsychiatric diseases hold promise in exploring the skin as a source of diagnostic, prognostic and therapeutic biomarkers in other diseases such as AID with psychiatric manifestations. Indeed, the skin is an accessible organ to perform biopsies. Moreover, less invasive skin investigations such as microbiopsies or even skin surface lipid–RNAs measures in sebum analysis have been recently developed (Lin et al., 2013; Shima et al., 2022). Single-cell transcriptomics of skin immune and non-immune cells can provide valuable insights into the molecular mechanisms underlying systemic and neuroinflammation and potentially lead to the discovery of new biomarkers (Jain et al., 2023). The utilization of "-omics" technologies such as proteomics and transcriptomics, allow for comprehensive analysis of various molecular components and pathways involved in AID. These technologies have the potential to revolutionize the field by providing a more precise and personalized approach to diagnosis and treatment.

### 1.4 Therapeutic implications

Current therapeutic strategies in AID include the use of colchicine (as the gold standard drug), immunomodulatory drugs, and biological agents targeting specific cytokines or immune cell receptors. Colchicine, a classical anti-mitotic drug, interferes with tubulin-related cellular processes, including inflammatory cytokine release and chemotaxis. It inhibits caspase-1 activation in a dose-dependent manner, thus reducing IL-1β and IL-18 production by immune cells (Gasparyan et al., 2015). New developments in immunobiology and genetics have also led to the development of innovative AID therapies (Ashari et al., 2023). The main therapies are summarized in Table I.

Anti-cytokine treatments are the main emerging therapeutic options for patients with AID; they can target different organs such as the skin and the brain (if capable of crossing blood-brain barrier). Patients suffering from AID caused by increased IL-1β signaling showed good response with anakinra, rilonacept (recombinant IL-1 receptor IL-1R that binds to IL-1α, IL-1β, and IL-1RA), and canakinumab (human monoclonal antibody that binds to IL-1β) (Georgin-Lavialle et al., 2019; Blackstone et al., 2023). Long-term efficacy and safety of these drugs are very promising as seen in a case report with cutaneous and inflammatory remission lasting 12 years post-follow-up in a child having Chronic Infantile Neurologic Cutaneous Articular Syndrome (CINCA) treated with anakinra (Costagliola et al., 2024). There is some evidence suggesting that anakinra may have better CNS penetration (Blackstone et al., 2023). It is well-known that patients with MDD and elevated pro-inflammatory biomarkers are often resistant to conventional antidepressants (Lauden et al., 2021)but immune-based therapies may help by targeting inflammation-related gene signatures (Drevets et al., 2022; Kaufmann et al., 2017). However, the effectiveness of immunotherapies modulating IL-1 signalling on depressive or anxiety symptoms remains to be determined by specific studies in this area.

For AID defined by abnormalities in the TNF pathway, off-label anti-TNF drugs are suggested in many case reports with clinical and biological improvement (Blackstone et al., 2023). Recently, TNF has been implicated in CINCA pathogenesis and successful treatment of two children with anti-TNF drug etanercept has shown clinical remission of cutaneous and neurologic symptoms as well as alleviation of systemic inflammation (Luo et al., 2020). Cytokine modulators may be novel drugs for MDD in chronically inflamed subjects, as anti-TNF-α such as infliximab improved MDD symptoms in patients with increased CRP levels (Raison et al., 2013). Ongoing clinical trials are assessing more precisely the effects of infliximab on measures of anhedonia, motivational behavior and circuitry and glutamatergic changes in the basal ganglia in patients with MDD (Kappelmann et al., 2018; Lee et al., 2018). Treatment with anti-TNF drug adalimumab in patients with hidradenitis suppurativa results in reduced pain scores and improved depressive symptoms (Scheinfeld et al., 2016).

In our experience as well as some reported cases, individuals resistant to anti-IL1 or anti-TNF therapies can benefit from IL-6 blocking therapies (Musters et al., 2015). Recent data show promising effects of anti-IL6 tocilizumab in inflammatory dermatoses and ongoing studies are assessing its efficiency in immune psychosis (Mastorino et al., 2022; Foley et al., 2023). Anti-IL18 drugs are under investigation in AID especially those associated with MAS or neurological/psychiatric manifestations with favorable safety profile and efficacy (Baggio et al., 2023)Koné-Paut and Piram, 2012 Ihim et al., 2022. In addition, anti-IL18 is thought to be a promising option to treat dermatologic manifestations associated with inflammasomopathies as an add-on or in case of insufficient response to anti-IL1 (Fenini et al., 2017). Furthermore, IL-1β and IL-18 have been implicated in stress-induced cutaneous diseases such as alopecia and targeting these cytokines can treat both psychiatric and dermatologic manifestations aggravated by psychological stress, which can be beneficial in the context of AID (Xiao et al., 2024). Inhibition of NLRP3 inflammasome and downstream cytokines is an emerging study topic in many animal models of stress induction, anxiety and depression further elucidating the PNI pathophysiology of psychiatric disorders (Yu et al., 2021; Liu et al., 2022). JAK inhibitors (JAKIs), such as baricitinib or ruxolitinib, can significantly improve quality of life and alleviate anxiety and depression symptoms, as demonstrated in various dermatologic conditions (Piraccini et al., 2023; Reguiai et al., 2023).

In the skin, mineralocorticoid receptor (MR) antagonists could be interesting therapeutic options for skin inflammation (Sevilla and Pérez, 2018). Furthermore, MR blockade prevents stress-induced memory impairment in rats and improves cognitive performance in patients with bipolar disorders (Deuter et al., 2024; Albernaz-Mariano and Demarchi Munhoz, 2022; Zandifar et al., 2023). Glucocorticoid receptor (GR) and MR modulators should further be assessed as interesting future therapeutic options for psychiatric as well as cutaneous manifestations of AID. Finally, many other drugs are still in research investigation such as developing drugs that specifically target TLRs or S100A12 proteins, which are recognized by innate immune system cells through TLR4 receptors (Dumur et al., 2023).

Interestingly, some antidepressant and antipsychotic molecules used for the treatment of psychiatric conditions have shown efficacy in decreasing systemic inflammation. It has been shown that SSRI treatment reduces TLR-mediated neuroinflammation as well as IL-6 mRNA expression in depressive patients further highlighting their anti-inflammatory potential (Pastis et al., 2024). For example, the antidepressant escitalopram used to treat a patient suffering from colchicine-resistant FMF with depressive symptoms improved other clinical symptoms such as fever and abdominal pain (Toshida et al., 2021). SSRIs can decrease the number of acute FMF attacks by increasing levels of the anti-inflammatory cytokines and reducing the proinflammatory ones (Lidor et al., 2021). Clinical studies show that antidepressants like fluoxetine, paroxetine, and others can induce autophagy and reduce the expression of NLRP3 inflammasome components and inflammatory cytokines IL-1β and IL-18 (Kouba et al., 2023).These molecules are further investigated in animal models as promising agents to treat immune-mediated disorders in many organs. In a rat model of chronic lung inflammation and fibrosis, mirtazapine, an atypical antidepressant downregulated NLRP3 inflammasome activity and repressed downstream IL-1β and IL-18 (Abdelhady et al., 2023). Thus, in the future, these molecules can be an ideal treatment option to alleviate not only psychiatric symptoms but also cutaneous and systemic manifestations in patients with AID, especially those resistant to classical anti-inflammatory treatments.

Therapeutic choices are still not standardized and must be discussed in a multidisciplinary approach. Interestingly, when patients respond to treatment we observe a global clinical improvement, further confirming the common immunological pathophysiology and the neuro-immune dialogue between different affected organs including the skin and the brain.

## 2 Conclusion

The hallmark of AID pathophysiology is a dysregulation of the innate immune system leading to disruption of physiological cross-talk between neuro-immuno-endocrino-cutaneous systems and leading to excessive deleterious inflammation. The skin-brain interaction, mainly mediated by cytokines and neuropeptides, plays an important role in AID. Through future clarification of the fundamental mechanisms entailed in this interaction, scientists might pinpoint new diagnostic and prognostic biomarkers as well as therapeutic targets for the management of AID in a more effective personalized approach. From the standpoint of PNI and immunopsychiatry, neurological and psychological variables may have an impact on innate immune system dysregulation and should be further assessed in patients with AID to offer a holistic therapeutic approach focusing on patients’ quality of life.

## CRediT authorship contribution statement

**S. Matar:** Writing – review & editing, Writing – original draft, Formal analysis, Conceptualization. **S. Aractingi:** Supervision, Conceptualization. **R. Gaillard:** Supervision, Conceptualization. **A.-C. Petit:** Writing – review & editing, Supervision, Methodology, Conceptualization.

## Patient consent on file

Consent for the publication of recognizable patient photographs or other identifiable material was obtained by the authors and included at the time of article submission to the journal stating that all patients gave consent with the understanding that this information may be publicly available.

## Funding sources

None.

## Declaration of competing interest

The authors declare that they have no known competing financial interests or personal relationships that could have appeared to influence the work reported in this paper.

## Data Availability

No data was used for the research described in the article.
